# Supplementary material for: NOCICEPTRA2.0 - A comprehensive ncRNA atlas of human native and iPSC-derived sensory neurons
Source: iScience. 2023 Nov 23;26(12):108525. doi: 10.1016/j.isci.2023.108525 (PMC10755718; doi:10.1016/j.isci.2023.108525)
Supplement: Document S1. Figures S1–S6 and Tables S1–S4 [file mmc1.pdf]

iScience, Volume *26*

Supplemental information

NOCICEPTRA2.0 - A comprehensive ncRNA atlas of human native and iPSC-derived sensory neurons

Maximilian Zeidler, Diana Tavares-Ferreira, Jackson Brougher, Theodore J. Price, and Michaela Kress

Supplementary Figures

Supplementary Figure S1: DGE of ncRNA throughout iPSC-derived sensory neuron development

Differential gene expression analysis of all ncRNAs. **(A)** Top 50 regulated ncRNAs including snoRNAs, lincRNAs, tRNAs, piRNAs, snRNAs, scaRNAs clustered rows into same trajectories and split by differentiation timepoints (columns). **(B)** Highly expressed ncRNAs were depicted as a function of the negative log10 p-adjusted value to determined highly significantly expressed ncRNAs

Related to: Time dependency of small RNAs throughout human iPSC-derived sensory neuron differentiation (Figure 1)

Supplementary Figure S2 Count frequency and hDRG analysis

(A) iDN sample-wise distributional analysis of count frequencies aggregated on the biotype per sample (B) hDRG sample-wise count frequence aggregated per biotype/sample. (C) Hierachical clustering of the correlation dissimilarity of all hDRG samples (D) PCA of hDRG samples from variance stabilized counts. (F) Expression trajectories of the hsa-let-7 family throughout development depicted using raw counts.

Related to: Time dependency of small RNAs throughout human iPSC-derived sensory neuron differentiation (Figure 1)

Supplementary Figure S3 Comparison hDRG and iPSC ncRNA expression

(A) Top-expressed ncRNA per biotype in hDRGs and (B) top-expressed ncRNAs per biotype in iDNs

Related to: Time dependency of small RNAs throughout human iPSC-derived sensory neuron differentiation (Figure 1)

Supplementary Figure S4 Specific ncRNA expression analysis of tRNAs

Analysis of tRNAs, mt-tRNAs and snoRNA host-genes. **(A)** tRAX was used to realign ncRNA counts to tRNAs and tRNA fragments only, since this pipeline is specialized for tRNA expression analysis. Precise location of tRNA reads were determined considering 5’ and 3’ or other fragments based on the location of the read**. (B)** Mitochondrial tRNA variance stabilized counts visualized as heatmap. **(C)** Correlation analysis of snoRNA host-genes with snoRNAs using Pearson correlation coefficient. **(D)** Expression patterns of the topmost negatively and positively correlated host-genes.

Related to: Differential temporal distribution of tRNA fragments during sensory neuron development (Figure 2)

Supplementary Figure S5 tRNA-miRNA target analysis

(A) Expression trajectories of tRNA-GlyCCC and tRNA-GluCTC using variance stabilized counts. Sankey plot of overlapping target-spaces between miRNAs and tRNAs indicating which miRNAs are most likely mimicked by the tRNA **(B)**. **(C)** Trajectories of mimicked miRNAs derived from NOCICEPTRA using variance stabilized counts

Related to: Differential temporal distribution of tRNA fragments during sensory neuron development (Figure 2, Figure 3)

Supplementary Figure S6 Cluster Analysis of sRNA and rRNAs

Clustering Analysis of sRNAs (A-B) and rRNAs (C-D)

Related to: Temporal regulation of rRNAs, snRNAs and piRNAs (Figure 5)

Supplementary Tables

Supplementary Table S1 hDRG Donor Table total RNA

Organ donor Table of hDRG samples (levels L1, L2, L3, L4, L5, T12) used to obtain Total RNAseq.

Related to: Experimental Model and Study Participant Details (Star Methods)

Supplementary Table S2 hDRG Donor Table small RNA

Organ donor Table for hDRG samples used to obtain miRNA with the NextFlex Kit.

Related to: Human native DRG analysis (Star Methods)

Supplementary Tables 3-4 Ontology Analysis snoRNAs

Enrichment Analysis of positively (Sheet 1) and negatively (Sheet 2) correlated snoRNA host-gene determined using g:Profiler ontology analysis.

Related to: Differential temporal distribution of snoRNAs in sensory neuron development (Figure 4)

Supplementary Figure S1

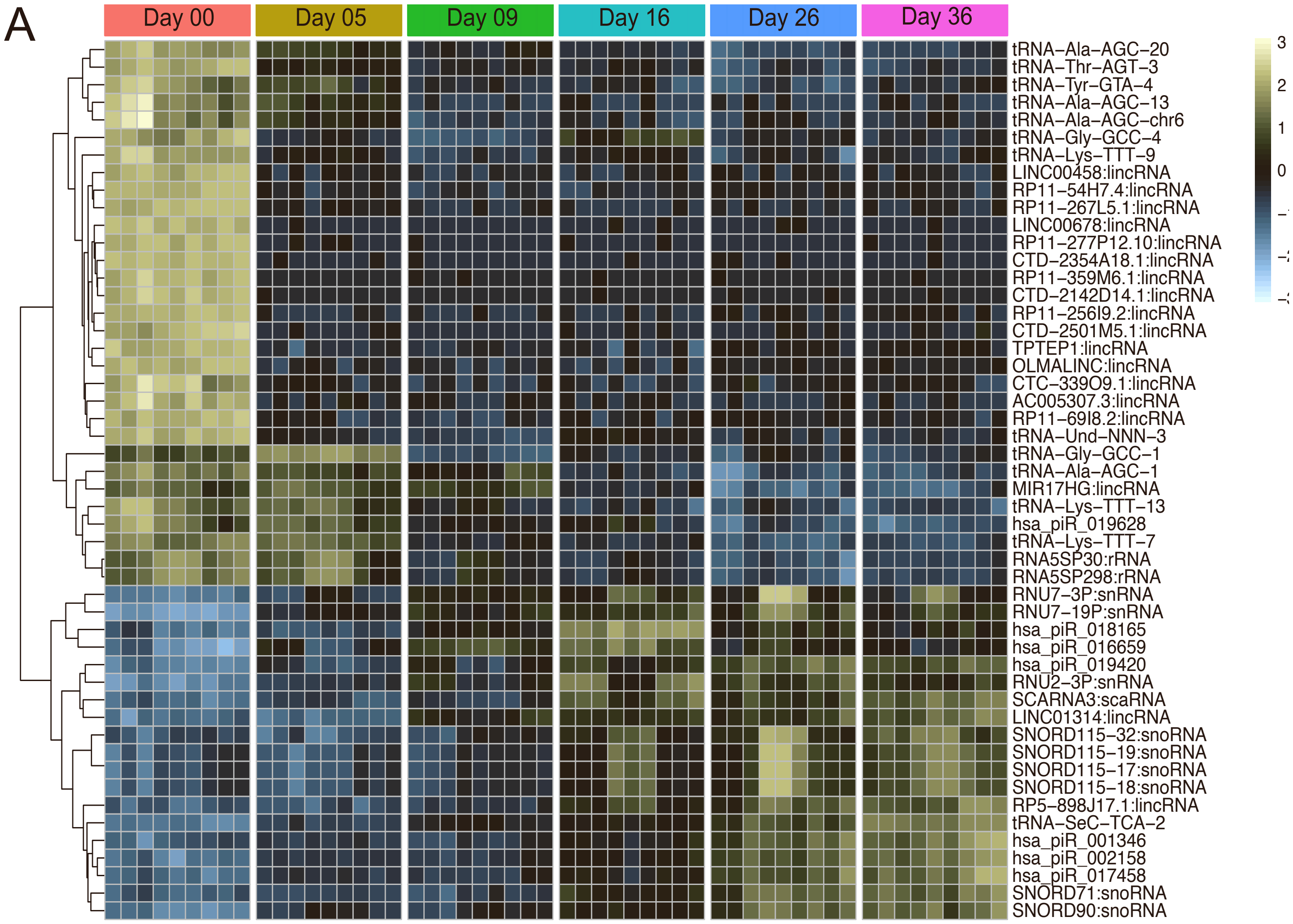

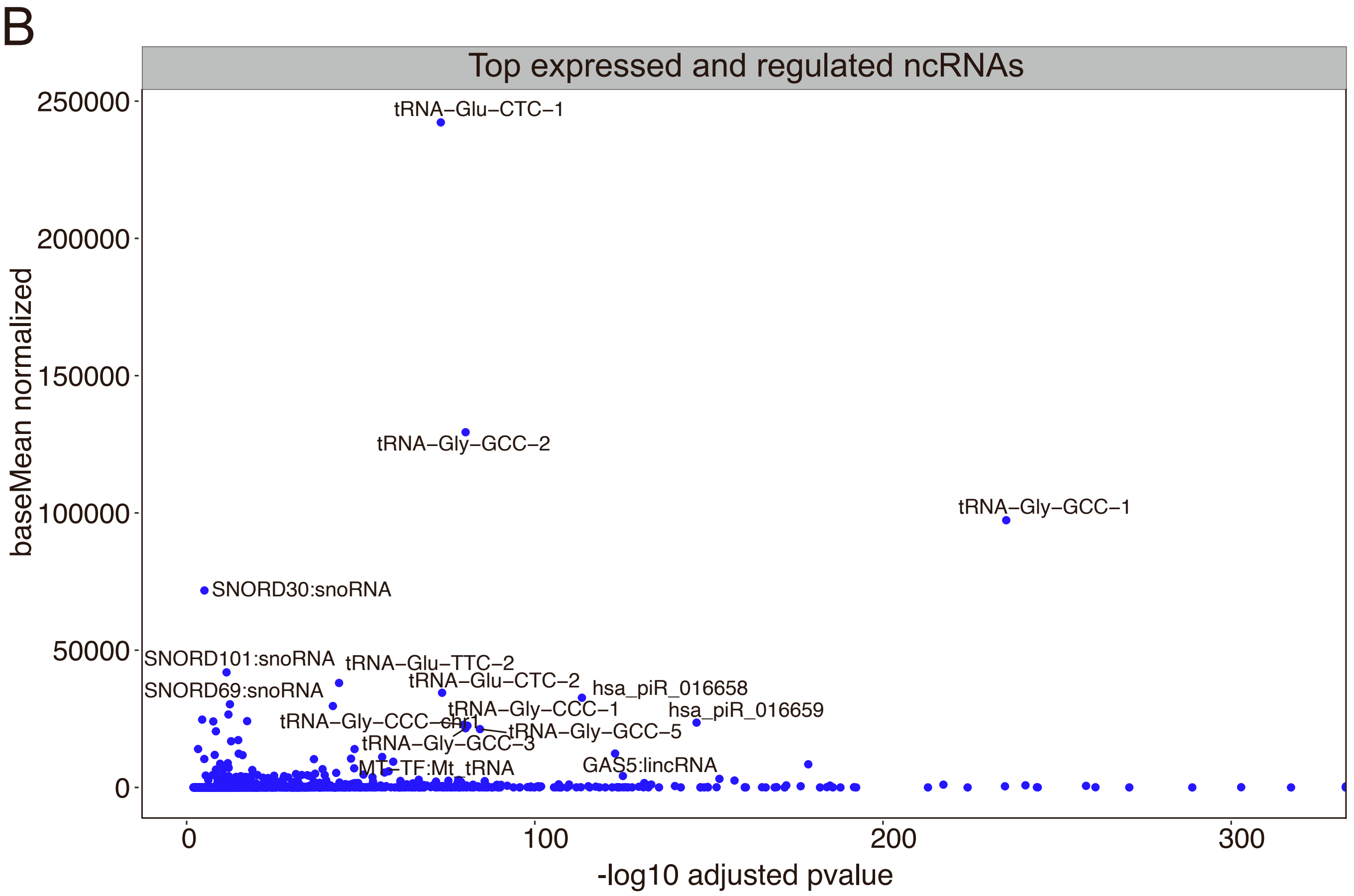

Supplementary Figure S2

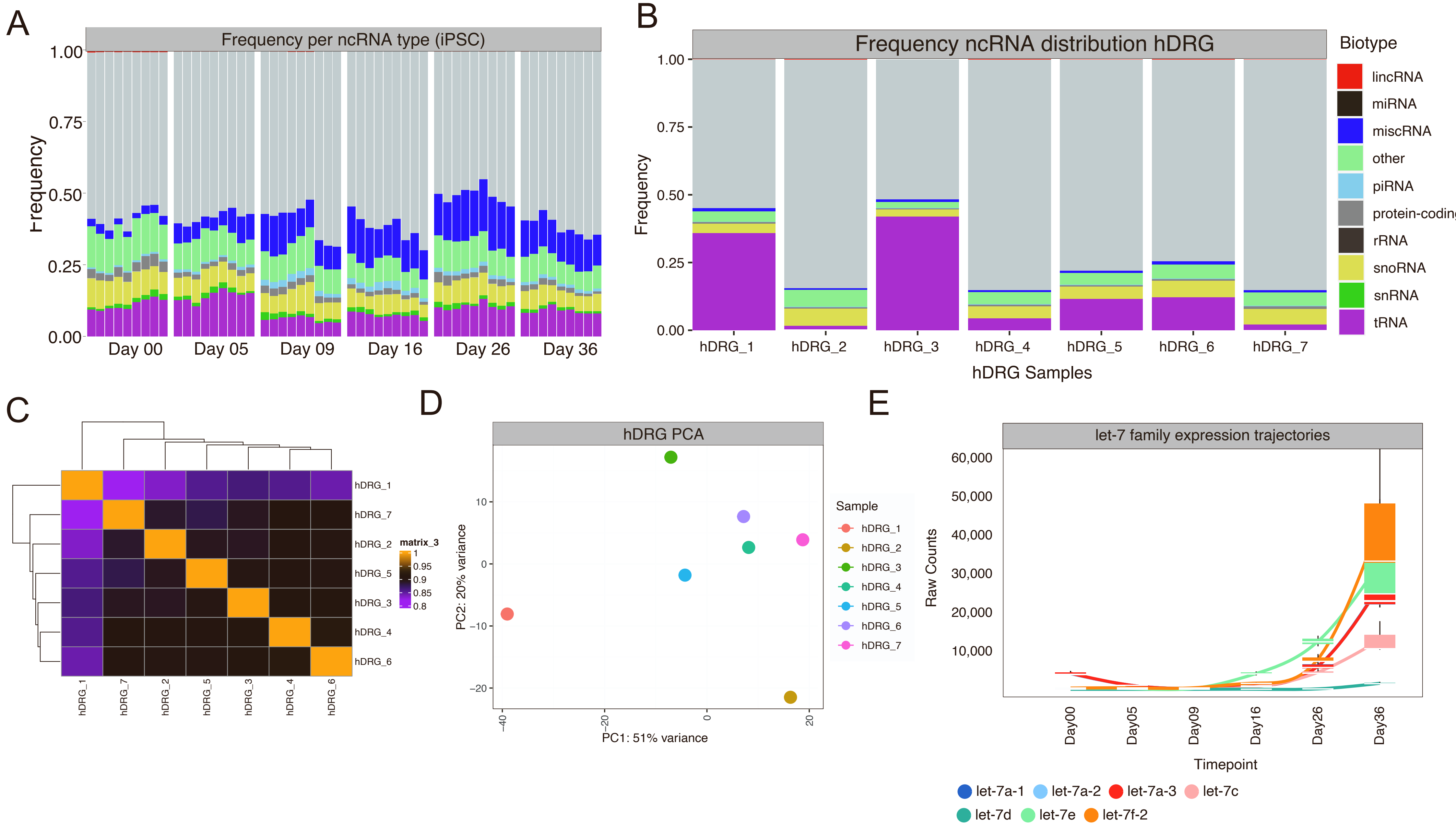

Supplementary Figure S3

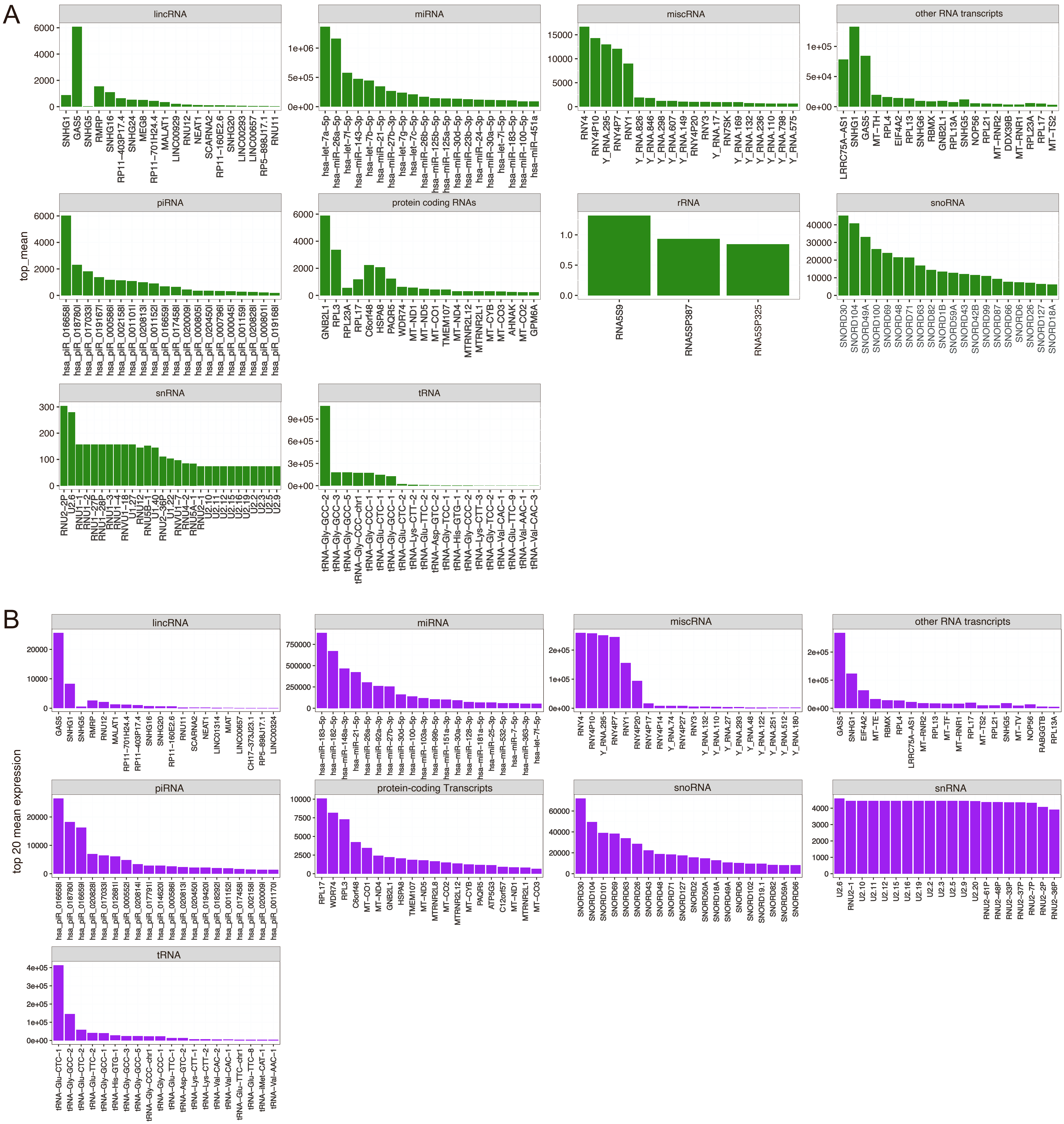

Supplementary Figure S4

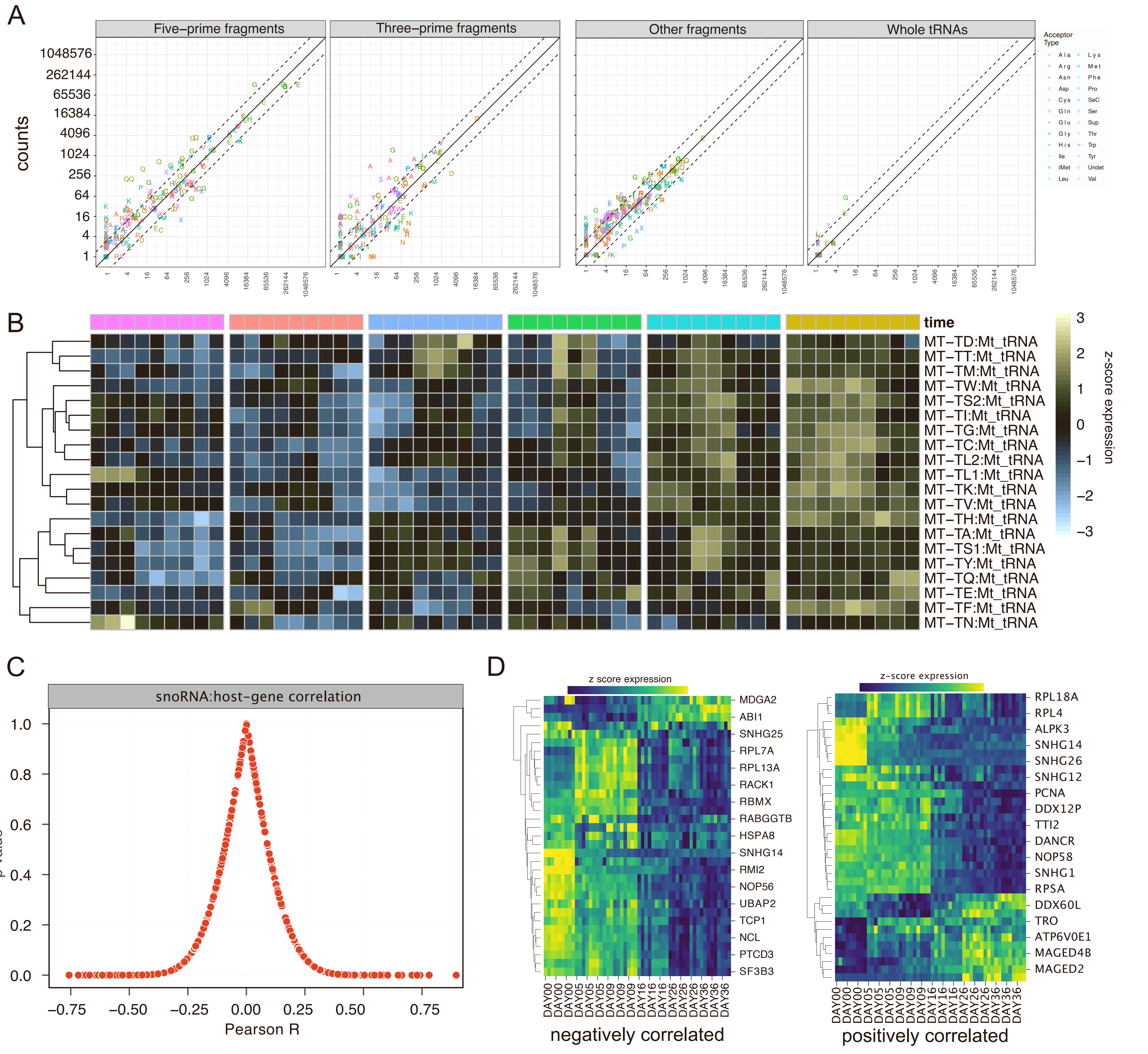

Supplementary Figure S5

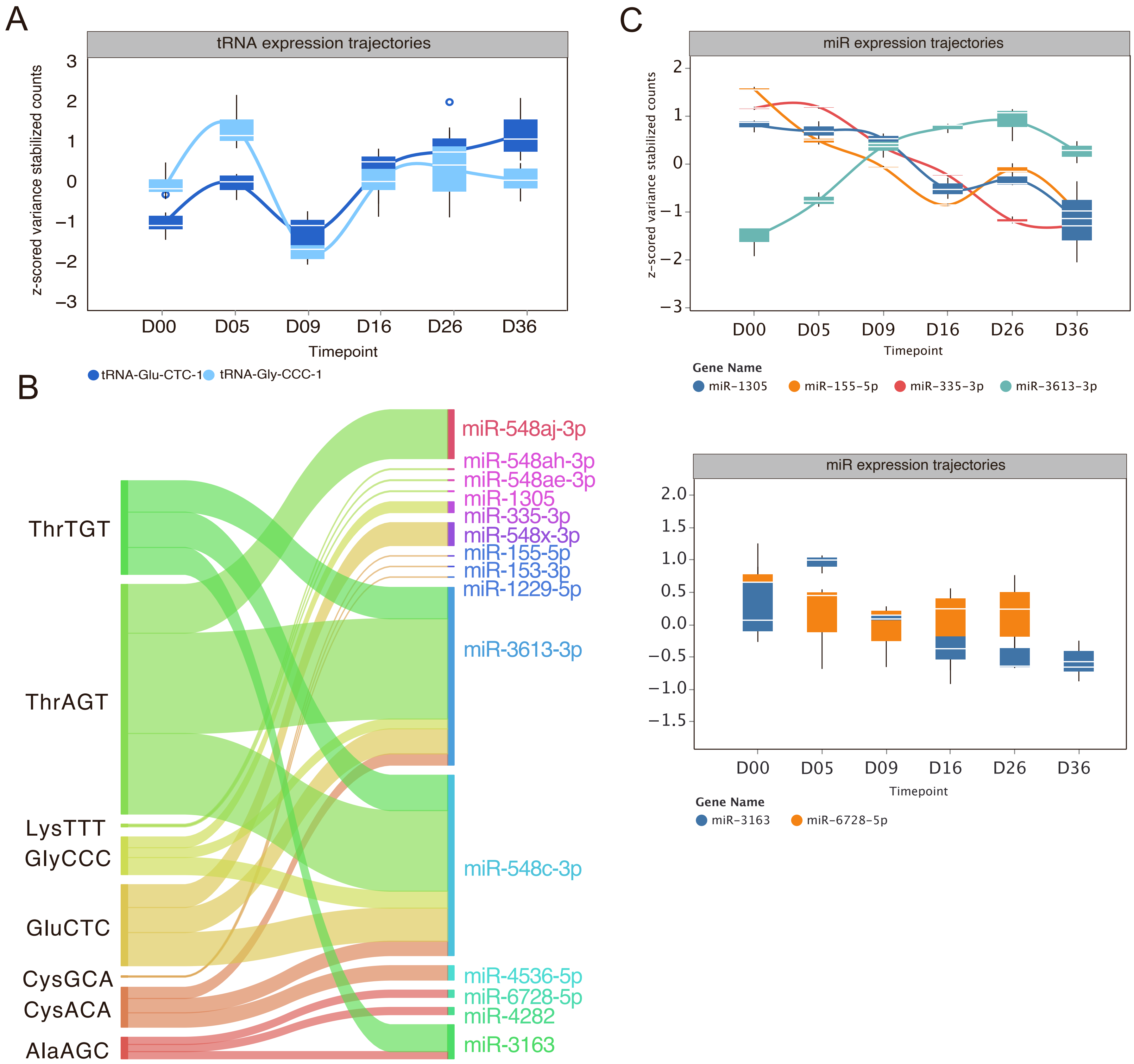

Supplementary Figure S6

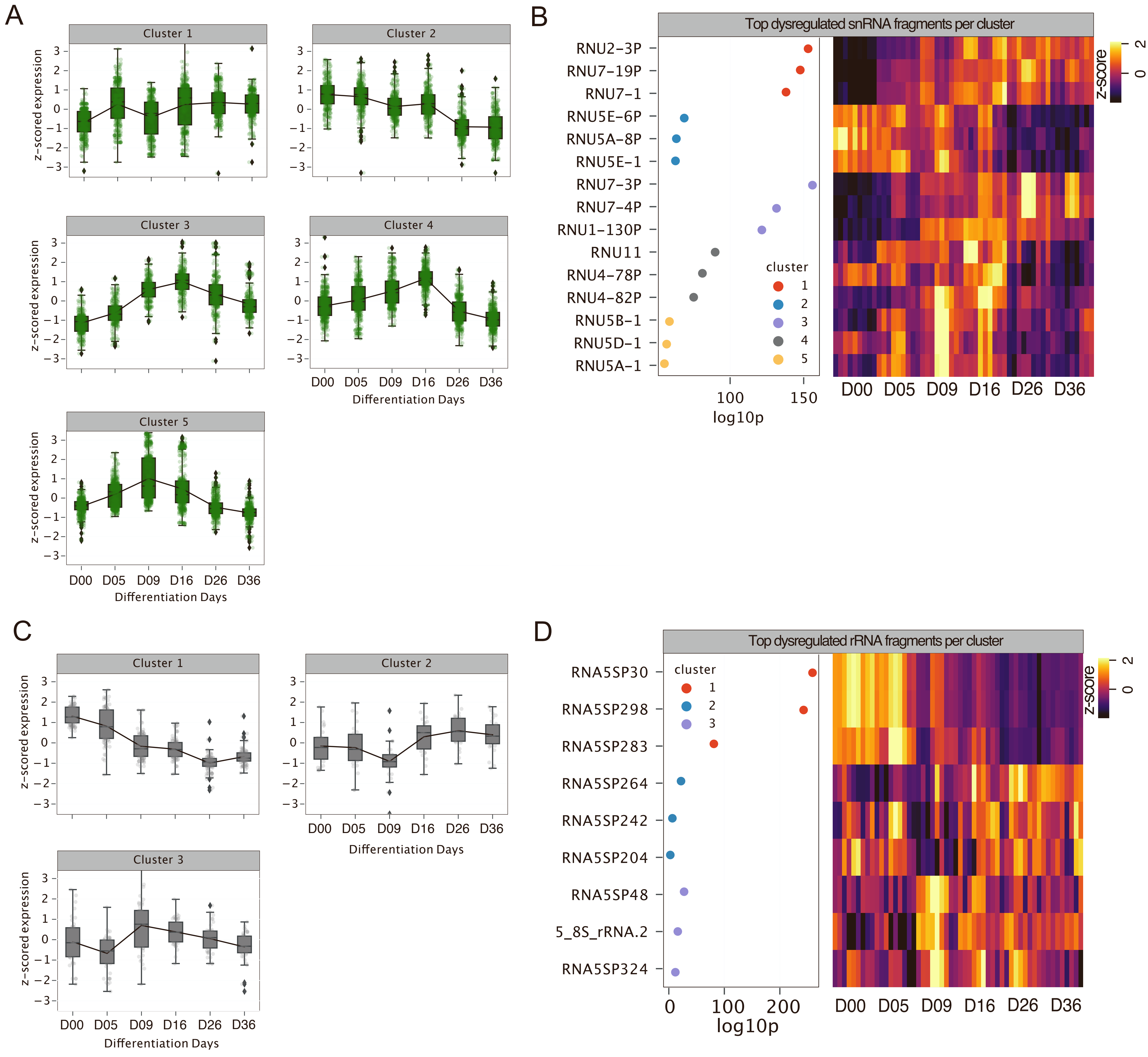

Supplementary Table S1

Donor ID	Age	Sex	Cause of death
**1**	33	F	Anoxia/Cardiac Arrest

Supplementary Table S2

Donor ID	Age	Sex	Ethnicity	COD
F1	58	F	White	Head Trauma/Falling off Golf Cart
F2	29	F	White	Anoxia/Drug Overdose
F3	75	F	White	CVA/Stroke
F4	73	F	Hispanic	CVA/Stroke
M1	34	M	White	CVA/Stroke
M2	30	M	Black	Anoxia/Asthma attack
M4	19	M	White	Anoxia/Drug Overdose

Supplementary Table S3

go-terms	description	source	p-value
GO:0005730	nucleolus	GO:CC	1,08E+10
GO:0022626	cytosolic ribosome	GO:CC	0.0023245640085109154
GO:0031428	box C/D RNP complex	GO:CC	0.0077738453767483996
GO:0070761	pre-snoRNP complex	GO:CC	0.0077738453767483996
GO:0034641	cellular nitrogen compound metabolic process	GO:BP	0.014697272672230416
GO:0006396	RNA processing	GO:BP	0.014971853624800283
GO:0032077	positive regulation of deoxyribonuclease activity	GO:BP	0.023495731002463938
GO:0044391	ribosomal subunit	GO:CC	0.024070215963835462
GO:0090304	nucleic acid metabolic process	GO:BP	0.03521732142300174

Supplementary Table S4

go-terms	description	source	p-value
GO:0005730	nucleolus	GO:CC	4,90E+07
GO:0002181	cytoplasmic translation	GO:BP	5,68E+10
GO:0045296	cadherin binding	GO:MF	1,11E+11
GO:0005681	spliceosomal complex	GO:CC	7,82E+10
GO:0022625	cytosolic large ribosomal subunit	GO:CC	0.00030951366040827915
GO:0019843	rRNA binding	GO:MF	0.0011190519802816948
GO:0003729	mRNA binding	GO:MF	0.005238960374892231
GO:0006396	RNA processing	GO:BP	0.013419434379018652
GO:0005654	nucleoplasm	GO:CC	0.02253047824430388
GO:0050685	positive regulation of mRNA processing	GO:BP	0.024708771478292972
GO:0043021	ribonucleoprotein complex binding	GO:MF	0.03385943442360295
GO:0006364	rRNA processing	GO:BP	0.038306978074175414
GO:0006417	regulation of translation	GO:BP	0.04315646699148754
GO:0030684	preribosome	GO:CC	0.04874749775200374
